# Early targeted patent ductus arteriosus treatment in premature neonates using a risk based severity score: study protocol for a randomised controlled trial (PDA RCT)

**DOI:** 10.12688/hrbopenres.13140.1

**Published:** 2020-11-25

**Authors:** Afif EL-Khuffash, Neidin Bussmann, Colm R. Breatnach, Aisling Smith, Elizabeth Tully, Joanna Griffin, Naomi McCallion, John David Corcoran, Elena Fernandez, Claudia Looi, Brian Cleary, Orla Franklin, Patrick J. McNamara

**Affiliations:** 1 Department of Neonatology, Rotunda Hospital, Dublin, Ireland; 2 Department of Paediatrics, Royal College of Surgeons in Ireland, Dublin, Ireland; 3 Department of Obstetrics & Gynaecology, Royal College of Surgeons in Ireland, Dublin, Ireland; 4 Department of Research & Academic Affairs, Rotunda Hospital, Dublin, Ireland; 5 Department of Pharmacy, Rotunda Hospital, Dublin, Ireland; 6 School of Pharmacy, Rotunda Hospital, Dublin, Ireland; 7 Department of Paediatric Cardiology, Our Lady's Children's Hospital, Crumlin, Dublin, Ireland; 8 Division of Neonatology, Stead Family Children’s Hospital Stead Family Children’s Hospital, Iowa, USA; 9 Departments of Pediatrics and Cardiology, University of Iowa, Iowa, USA

**Keywords:** Patent Ductus Arteriosus, Randomised Controlled Trial, Ibuprofen, Severity Score

## Abstract

A patent ductus arteriosus (PDA) in preterm infants is associated with increased ventilator dependence and chronic lung disease, necrotizing enterocolitis, intraventricular haemorrhage, and poor neurodevelopmental outcome. Randomised controlled trials of early PDA treatment have not established a drop in the aforementioned morbidities. Those trials did not physiologically categorise PDA severity. Incorporating the specific physiological features of a haemodynamic significant PDA may evolve our understanding of this phenomenon, allowing accurate triaging using echocardiography and targeted treatment.  Our group has recently demonstrated that a PDA severity score (PDAsc) derived at 36-48 hours of age can accurately predict the later occurrence of chronic lung disease or death (CLD/Death). Using echocardiography, we assessed PDA characteristics, as well as left ventricular diastolic function and markers of pulmonary overcirculation, and from this formulated a PDAsc. Gestation was also incorporated into the score. We hypothesise that in preterm infants at high risk of developing CLD/Death based on a PDAsc, early treatment with Ibuprofen compared with placebo will result in a reduction in CLD/Death. This is a single centre double-blind two arm randomised controlled trial conducted in the neonatal intensive care unit in the Rotunda Hospital, Dublin. Echocardiogram is carried out in the first 36-48 hours of life to identify preterm infants with a PDAsc ≥ 5.0 and these infants are randomised to Ibuprofen or placebo. Primary outcomes are assessed at 36 weeks post menstrual age. This pilot study’s purpose is to assess the feasibility of performing the trial and to obtain preliminary data to calculate a sample size for a definitive multi-centre trial of early PDA treatment using a PDAsc. We aim to recruit a total of 60 infants with a high risk PDA over three years.

**Trial Registration: **ISRCTN ISRCTN13281214 (26/07/2016) and the European Union Drug Regulating Authorities Clinical Trials Database 2015-004526-33 (03/12/2015).

## Introduction

The ductus arteriosus (DA) links the pulmonary artery to the descending aorta and is a key part of normal fetal circulation. After delivery the lungs expand and become the main site for oxygen exchange. At this stage closure of the DA will usually occur as it is no longer required. However, if the DA remains open following birth, especially in the premature population, it is associated with important pathological effects. It is the most common cardiovascular abnormality that affects premature infants. It occurs in about 30% of infants below 30 weeks gestation and up to two thirds of infants less than 28 weeks ^1^. A patent ductus arteriosus (PDA) is associated with the following pathologies in premature babies: increased mortality, worsened neurological outcome, necrotising enterocolitis (NEC), intraventricular haemorrhage (IVH), and chronic lung disease (CLD) ^2– 7^.

The timing of treatment of a PDA (within the first 72 hours of life) and the selection of infants most likely to benefit from treatment remain controversial topics ^8^. Randomised controlled trials investigating the treatment of a PDA have failed to illustrate a reduction in morbidities associated with a PDA ^9– 15^. Those trials were limited by a failure to physiologically categorise PDA severity and likely shunt volume; rather the selection of PDA cases was based on arbitrary dimensional criteria or less specific clinical criteria. Whether trials to date have ever treated the “actual” population of interest is debatable.

When referring to the term “haemodynamic significance” we should consider the volume of the shunt across the ductus as causing an increase in pulmonary blood flow (causing pulmonary overcirculation) with the secondary effect of systemic hypoperfusion. We should account for the extent of this shunt as well as its impact on the pulmonary and systemic circulation’s. A more detailed evaluation using echocardiography of those physiological features may improve our understanding of a haemodynamically significant PDA and enable more accurate targeted treatment.

The ductus arteriosus plays an important role during the transition from intra-uterine life in the premature babies’ early hours and as such it should be viewed as a beneficial physiological component in aiding the reduction of pulmonary hypertension and supporting the right ventricle, as pulmonary vascular resistance drops. However, persistent patency of the ductus in conjunction with reducing pulmonary pressures, can lead to either systemic hypoperfusion and pulmonary congestion or chronic pulmonary hypertension due to the long term exposure of the pulmonary vascular bed to systemic vascular pressures. Defining the ductus once it reaches haemodynamic significance is challenging. Both clinical and echocardiography measures have been used to define haemodynamic significance. But these are not consistent, and can lack clear validation ^16^. Clinical features utilised to identify these ducts may even include short term clinical signs such as a murmur, an active praecordium or bounding pulses ^17^. 

Recently trials of PDA treatment have attempted to relate PDA associated morbidities, such as chronic lung disease with the patent ductus. Certainly, in trials involving the baboon population continued ductal exposure with secondary increased pulmonary blood flow led to impaired lung function in this population. Furthermore, it was noted that alveolar development ceased and alveolar surface area was reduced. Medical closure of the PDA in the baboon population essentially reversed the pulmonary effects and improved lung function ^18^. Sehgal *et al.* used a score of PDA significance using echocardiography markers at a median of 7 days of age and demonstrated an association with CLD in premature babies ^19^. Although cause could not be directly inferred, Sellmer *et al.* illustrated that large PDA’s identified on day 3 of life were associated with CLD, mortality and IVH ^20^. Another study, also utilising a PDA scoring system, identified babies who were exposed to a severe PDA for a longer duration during their admission were greater inclined to develop CLD ^21^. Furthermore, after adjusting for PDA severity, PDA ligation was no longer associated with CLD in Schena *et al.*’s cohort despite earlier trials by Clyman *et al.* showing an association between prophylactic PDA ligation within 24 hours of age and CLD. PDA severity was not accounted for in Clyman *et al.*’s trial ^22^. In recent studies, there is a greater acknowledgement for the lack of association between PDA ligation once the severity of the PDA is accounted for 23, 24.

Guyatt *et al.* identified a “need for a discriminative and predictive score” enabling identification and quantification of a haemodynamically significant PDA within the first 2 days of age ^25^. This score should identify premature infants with a significant ductus at risk of developing PDA associated morbidities and may be used to identify PDAs that warrant treatment and subsequently assessed in a randomised clinical trial setting. Gestational age should be included in the score as it is independently associated with morbidities similar to that of the PDA. Finally, the score should include echocardiography measures to quantify shunt size and volume. A recent editorial by our group discusses the three key components in the assessment of haemodynamic significance of a ductus. These factors included (1) assessment of shunt volume, (2) determination of myocardial function, and (3) identification of perinatal and antenatal modifiers. The determinant of shunt volume in a vessel whose length, pressure across the vessel, and blood viscosity is always changing, is hard to establish. Echocardiographic surrogate measurements of shunt volume such as markers of pulmonary over-circulation or systemic hypoperfusion can be used as estimates of true shunt volume ^26^. Attention should also be focused on left heart diastolic function. The left ventricle (LV) plays an important role in managing the higher blood volume returning to the heart secondary to a PDA. However, the premature infant has a less compliant myocardium than term infants causing impaired diastolic function and a greater dependence on the late diastolic phase of atrial contraction for ventricular filling ^27^. In the setting of higher LV preload, impaired diastolic function can lead to raised left atrial pressure and subsequent pulmonary venous congestion ^28^. Incorporating echocardiography measures of LV diastolic function into an early score of haemodynamic significance will better identify a truly pathological PDA. There is certainly a need to investigate the incorporation of myocardial function into a severity score when assessing a ductus in a preterm infant.

Our group devised a PDA severity score (PDAsc) based on data from a multicentre prospective observational study ^29^. The babies had a mean (SD) gestation of 26 (1.4) weeks and a mean (SD) birthweight of 952 (235) grams. Echocardiography was performed between 36-48 hours of age on 141 premature infants. Using markers of pulmonary overcirculation (left ventricular output) and left ventricular function (LV a` wave) identified on echocardiogram, a PDAsc was devised which could predict chronic lung disease or death before discharge (CLD/Death) in patients with a PDA. The score also included the infants PDA diameter and flow velocity, and their gestation at birth. All of the five parameters utilised for the score were identified using univariate analysis and multi logistic regression. Infants scores could range from 0 (low risk) to 13 (high risk). Babies who developed CLD/Death scored significantly higher [7.3 (1.8) vs. 3.8 (2.0), p<0.001]. A cut off PDAsc of 5.0 had an area under the curve (AUC) of 0.92 (95% CI 0.86 – 0.97, p<0.001) for ability to predict CLD/Death. A PDAsc cut off of five has sensitivity and specificity of 92% and 87% respectively, and positive and negative predictive values of 92% and 82%. Using this score, which identifies those infants at highest risk of developing PDA-associated morbidities such as CLD and death, could be critical in identifying high risk patients for the setting of a randomised controlled trial.

### Rationale for the study

PDA is a common cardiac condition in preterm neonates and leads to a significant burden of illness and is associated with increased mortality, and adverse neurodevelopmental outcome ^2– 7^. There is poor understanding of the physiologic impact of a DA that remains patent. Monitoring response to treatment in the intervention arm and observing the outcome in the control arm in patients identified using the risk based score will provide evidence both on the pathophysiology of a PDA and the efficacy of early targeted therapy.

This study is aimed to address the current knowledge gap in PDA treatment. The current failure of all RCTs to date is the failure to accurately identify the “at risk population” prior to treatment. A positive outcome in this pilot study will pave the way for a definitive trial of targeted PDA treatment. Reducing PDA-associated complications such as NEC and CLD will have substantial short and long term benefits for the infants, their families and the healthcare service. Reduced length of hospital stay will significantly reduce hospital costs in the neonatal intensive care unit. Ongoing management of NEC and CLD in the short and longer term poses a huge financial burden on the health service. In addition, NEC and CLD increase the risk of adverse neurodevelopmental outcome with all its associated ongoing financial and quality of life costs.

CLD is an extremely serious complication of premature delivery associated with high mortality rates both before and after discharge. In addition, CLD is associated with adverse neurodevelopmental outcome, poor growth and significant respiratory morbidity, with reduced lung function demonstrated into adulthood ^30^. NEC affects long term bowel function (including the presence of a stoma, admissions for bowel related problems and continued medical and surgical care) as well as impacting on motor, sensory and cognitive outcomes ^31^.

Ibuprofen (Pedea 5mg/ml), a non-steroidal anti-inflammatory drug (NSAID) is the current licenced medical option for early PDA closure. It is a non-selective COX inhibitor which causes a reduction in circulating prostaglandin levels, thereby promoting DA closure. Compared to indomethacin (its predecessor), Ibuprofen does not significantly reduce mesenteric and renal blood flow velocity, and causes less cerebral blood flow disturbances ^32, 33^. It also has a lesser effect on urinary anti-diuretic hormone excretion translating to fewer effects on renal function ^34^. Ibuprofen is as effective as indomethacin in closing a PDA and currently appears to be the drug of choice ^35^.

It is important to mention that both arms of this study are regarded as accepted approaches to PDA management. The use of Ibuprofen in PDA treatment is an accepted and approved approach. Ibuprofen is licenced for PDA treatment in Europe and North America. The purpose of this pilot study is to assess the feasibility of performing the trial and to obtain preliminary data to calculate a sample size for a definitive multi-centre trial of early PDA treatment using a PDAsc.

### Primary study objective

We aim to identify infants at high risk of developing CLD/Death by utilising the PDAsc, and randomise those infants to early treatment with Ibuprofen versus placebo. We hypothesise that in preterm infants less than 29 weeks gestation at high risk of developing CLD/Death based on a PDAsc ≥ 5.0 obtained using echocardiography carried out between 36 and 48 hours of life early treatment with Ibuprofen compared with placebo will result in a reduction of CLD/Death by 36 weeks post menstrual age (PMA). Infants with a PDAsc < 5 will not be enrolled in the study but will be followed up to discharge to confirm their low risk status.

### Secondary study objective

We will collect information on important secondary outcomes; sepsis (culture positive); NEC identified on Plain Film Abdomen with evidence of pneumatosis intestinalis; intraventricular haemorrhage assessed at 7 days of age; medication use including inotropic support, diuretic use and postnatal steroid administration; days on oxygen, non-invasive ventilation and invasive ventilation; hospital days; retinopathy of prematurity; periventricular leukomalacia.

### Exploratory objective

In this pilot study we also aim to ascertain issues with recruiting infants and obtaining consent, compliance with the protocol, the rate (if any) of loss to follow up, and the general acceptability, feasibility and compliance of administering the intervention. We aim to obtain preliminary data to calculate a sample size for a definitive multi-centre trial of early PDA treatment using a PDAsc. We aim to recruit a total of 60 infants, from a single neonatal centre over a three year period, with a high risk of developing PDA-associated morbidities. We then plan to seek additional funding under the EU Horizon 2020 scheme in order to facilitate the running of the multicentre RCT in European and North American centres.

## Methods: Participants, interventions, and outcomes

### Study design

**Figure 1.**
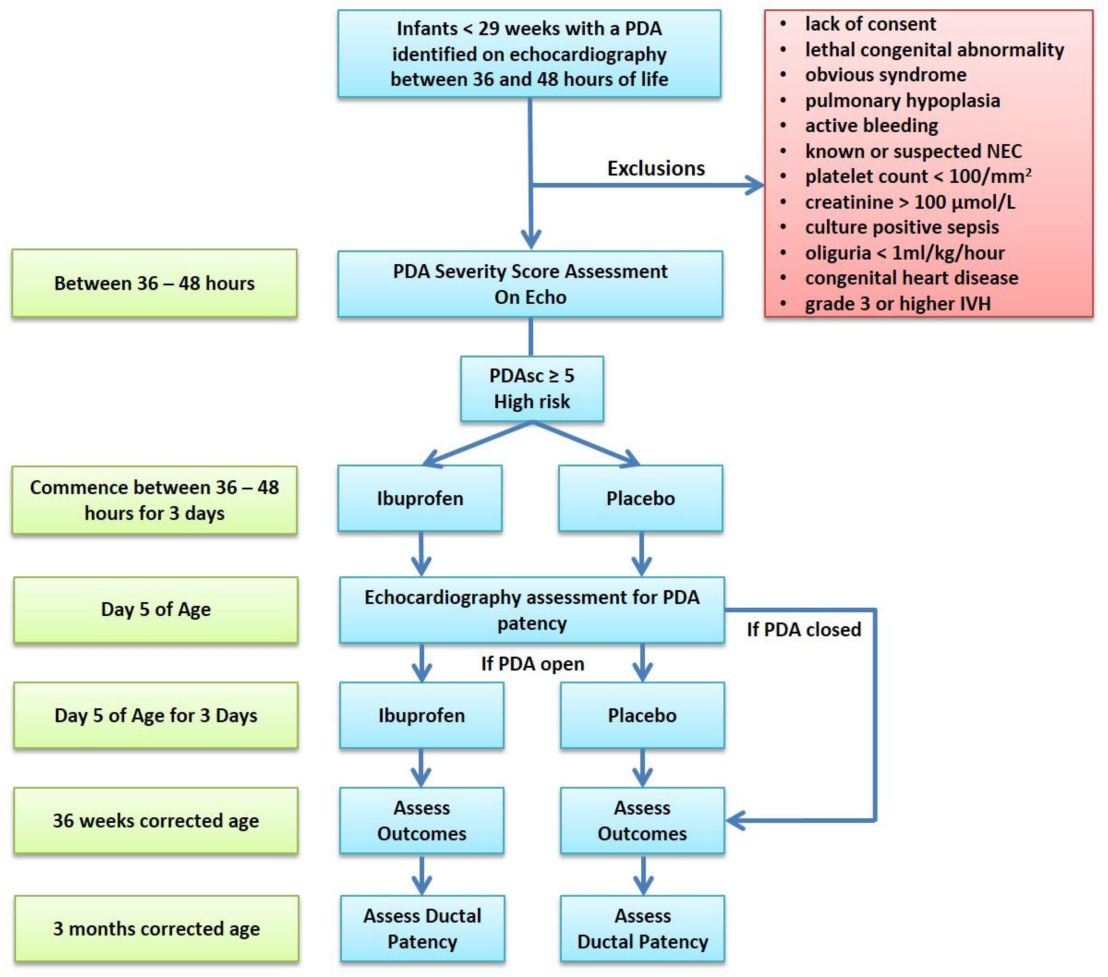
Study schematic. PDA = Patent ductus arteriosus; ECHO = echocardiogram; Nec = necrotising enterocolitis; IVH = intraventricular haemorrhage.

This is a single centre, randomised, double-blind, two arm pilot study, with a balanced (1:1) allocation that will be carried out in the level III neonatal intensive care unit (NICU) in The Rotunda Hospital Dublin. Our unit currently adopts a conservative approach to PDA treatment. During an infant’s first 14 days of age, there is no routine use of either prophylactic indomethacin or medical management of a PDA with Ibuprofen. Treatment beyond this is usually driven by ventilator dependence and the decision to treat is made by the attending physician caring for the infant. Hypotension that requires treatment during the study period is managed using local guidelines using a combination of both fluid resuscitation and inotropic support. A schematic of the study process is provided as Figure 1.

### Eligibility criteria

All infants less than 29 weeks at delivery admitted to the NICU with a PDA identified on echocardiography between 36 and 48 hours of life will be eligible for inclusion.

***Inclusion criteria*.** A comprehensive assessment of PDA significance will be performed using echocardiography between 36 and 48 hours of age to derive a PDA risk score based on this following formula: (Gestation *in weeks* × -1.304) + (PDA diameter *in mm* × 0.781) + (Left ventricular output *in ml/kg/min* × 0.008) + (maximum PDA velocity *in m/s* × -1.065) + (LV a` wave *in cm/s* × -0.470) + 41, where 41 is the constant of the formula. Infants with a risk score ≥ 5.0 are deemed to be at high risk of developing CLD/Death and will be randomised to either arm.

***Exclusion criteria*.** Infants are excluded from the trial if parents do not consent to the trial or there is a lack of availability of study investigators to carry out echocardiographic examination. Babies with a lethal congenital abnormality or an obvious syndrome, with structural congenital heart disease other than a PDA or PFO (patent foramen ovale), or those diagnosed with pulmonary hypoplasia are not eligible for inclusion. If there is suspected or known NEC, infants are also deemed ineligible. Other exclusion criteria include thrombocytopenia (platelet count < 100/mm ^2^), impaired renal function (creatinine count > 100 µmol/L; and/or oliguria < 1ml/kg/hour), culture positive sepsis, and active bleeding including a grade 3 or higher IVH or gastrointestinal haemorrhage. A grade 3 intraventricular haemorrhage is classified as bleeding within the ventricle and extension into the lateral ventricle with associated enlargement of the ventricle. A grade 4 haemorrhage is classified as an intraparenchymal haemorrhage ^36^.

### Interventions

***Intervention arm*.** Infants in the intervention arm will receive intravenous Ibuprofen (Pedea 5mg/1ml) at a dose of 10mg/kg (2ml/kg), followed by 2 doses of 5mg/kg (1ml/kg) 24 hours apart administered as a short infusion over 15 minutes. The patency of the ductus will be assessed 24 hours after the last Ibuprofen dose using echocardiography. If the PDA remains open (PDA diameter > 1.5 mm), then a second course of Ibuprofen will be given. No further doses of Ibuprofen will be administered.

***Control arm*.** Infants in the control group will receive an intravenous dose of placebo (normal saline) at a volume equivalent to that in the intervention group (2ml/kg 1 ^st^ dose; 1ml/kg 2 ^nd^ & 3 ^rd^ doses) administered as a short infusion over 15 minutes. The patency of the ductus will be assessed 24 hours after the last placebo dose using echocardiography. If the PDA remains open (PDA diameter > 1.5 mm), then a second course of placebo will be given. No further doses of placebo will be administered.

***Additional therapy and exit criteria*.** In keeping in line with the current practice in our unit, treatment of a PDA does not occur within the first two weeks of age and open label treatment beyond two weeks of age is at the discretion of the attending clinician. Those infants recruited in the trial who received open label treatment, will be treated with intravenous paracetamol (15mg/kg QDS for 3 days followed by 10mg/Kg QDS for two days). Infants who fail to close a PDA following paracetamol will be considered for PDA ligation ^37^. If any of the exclusion criteria occur a randomised baby within 1–3 days of administration of the study drug requiring discontinuation of the study drug, with return to standard care, these are considered as potential side-effects of Ibuprofen administration.

***Assessment of compliance*.** The investigator is responsible for ensuring that the study treatment is administered in compliance with the protocol. Compliance of administration will be checked by the study investigator on a daily basis (every dose) by consulting dispensing records and the infant’s drug chart for a signed, dated and timed record of drug administration.

***Overdose of study treatment*.** The following procedures will be conducted if an overdose of the study drug has occurred:

- Emergency unblinding and withdrawal from the study
- Urine output assessment hourly for 3 days
- Blood pressure assessment hourly for 3 days
- Heart rate assessment hourly for 3 days
- Assessment of feeding intolerance and hold feeding for 24 hours
- Assessment of gastrointestinal haemorrhage daily
- Full blood count for platelet and neutrophil assessment daily for 3 days
- Renal function including sodium and creatinine daily for 3 days

Fluid restriction will be carried out if there is significant oliguria (urine output less than 1ml/kg/hour). The renal team in Temple Street Children’s University Hospital will be consulted if there is evidence of acute renal failure. The haematology team in Our Lady’s Children’s Hospital, Crumlin will be contacted if there is severe neutropenia and/or thrombocytopenia or any active bleeding after emergency replenishment of platelets and other blood products as necessary.

***Prior and concomitant therapy*.** Any medication, other than the study medication taken during the study (from Subject consent to Hospital discharge) will be recorded in the CRF. Medication administration will be recorded as NICU protocols. 

The following drugs will not be used during Ibuprofen treatment. Treatment with Ibuprofen will discontinue and will not be recommenced if the use of any of the following medication is necessary:

- Diuretics: Ibuprofen may reduce the effect of diuretics; diuretics can increase the risk of nephrotoxicity of NSAIDs in dehydrated patients.
- Anticoagulants: Ibuprofen may increase the effect of anticoagulants and enhance the risk of bleeding.
- Corticosteroids: Ibuprofen may increase the risk of gastrointestinal bleeding.
- Nitric oxide: Since both medicinal products inhibit platelet function, their combination may in theory increase the risk of bleeding.
- Aminoglycosides (including gentamicin): since Ibuprofen may decrease the clearance of aminoglycosides, their co-administration may increase the risk of nephrotoxicity and ototoxicity.

### Outcomes

The primary composite outcome of chronic lung disease (CLD) or death before discharge is assessed at 36 weeks corrected gestational age (CGA). Chronic lung disease is defined as the need for oxygen supplementation at 36 weeks CGA ^38^.

We also monitor for the occurrence of the following secondary outcomes; pulmonary haemorrhage (defined as frank blood in the endotracheal tube without evidence of trauma), PDA ligation, culture positive sepsis, most recent cranial ultrasound findings assessing for intraventricular haemorrhage graded by the Papile classification ^36^ and periventricular leukomalacia as defined by Inder *et al.* ^39^, medical or surgical management of necrotising enterocolitis based on the Bell’s criteria (Bell), retinopathy of prematurity requiring laser treatment, and invasive or non-invasive ventilation history (a day on each modality is defined as more than 12 hours in a 24 hour period). We will also monitor for the administration of postnatal steroids, furosemide and inotropes, and the duration of hospital admission

### Participant timeline

All preterm babies less than 29 weeks gestation, admitted to the Rotunda NICU, are assessed for eligibility. All babies with a PDA, identified on comprehensive echocardiography between 36 and 48 hours of age, with a PDAsc ≥ 5.0 are deemed eligible for inclusion. All babies with a score <5.0 are not randomised to an intervention, but are followed to discharge in a similar fashion as the randomised infants. Infants are excluded if they meet one or more of the exclusion criteria.

**Figure 2.**
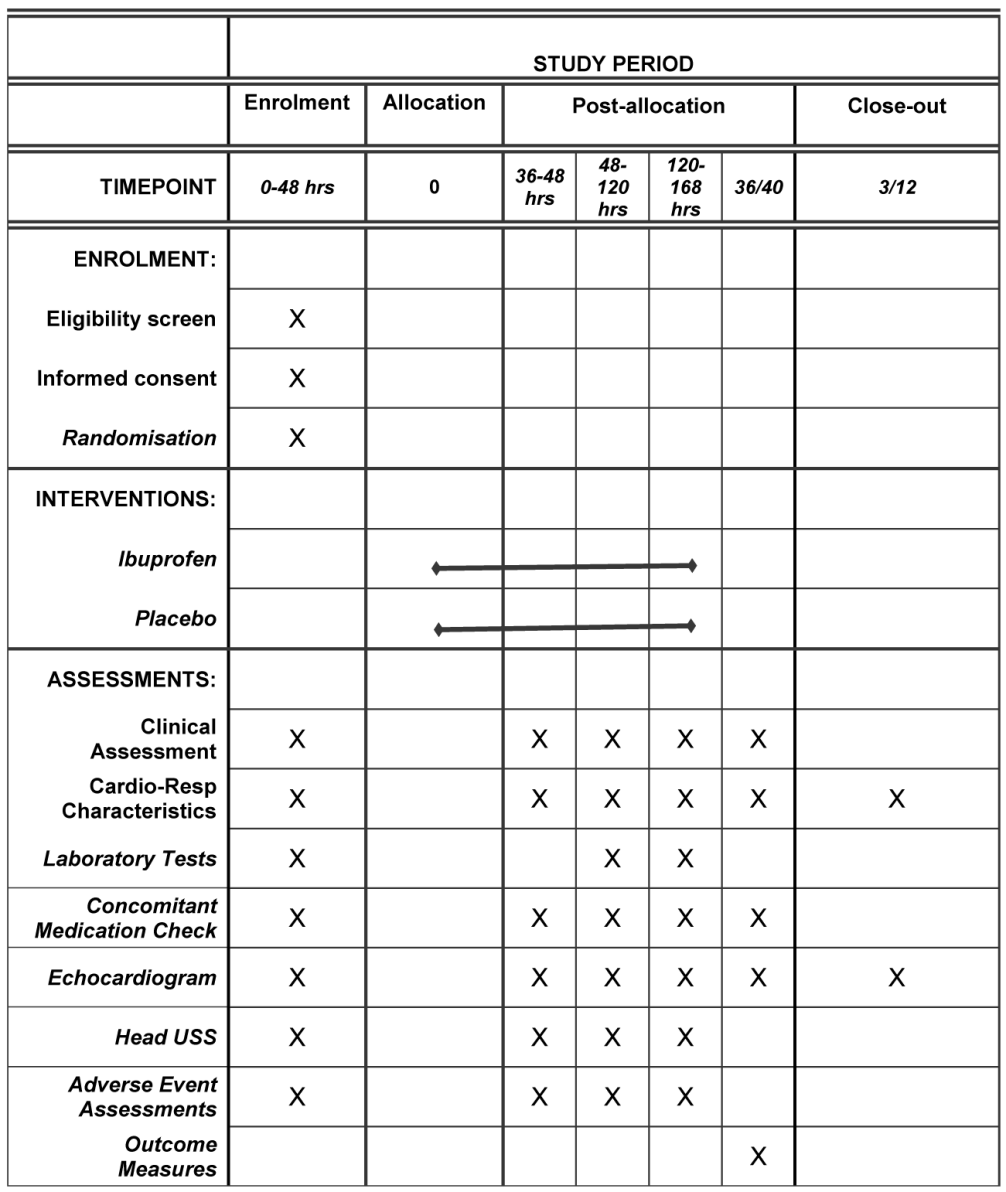
A schematic diagram detailing the participant timeline from enrolment to study closure. USS = ultrasound scan.

Antenatal, birth details and clinical characteristics are collected for all study participants. The antenatal details recorded within the first 48 hours of age include intrauterine fetal growth restriction, pre-eclampsia, premature rupture of membranes, multiple pregnancy, magnesium sulphate and steroid administration (none/one dose/two doses). We record surfactant administration, gestational age (weeks), birthweight (grams), sex, mode of delivery (vaginal delivery/caesarean section), 5-minute Apgar scores and cord pH. Clinical characteristics are recorded at each echocardiography timepoint and include details of cardiorespiratory stability, ventilation support, relevant co-treatments, laboratory results and fluid status. A cranial ultrasound scan is also performed prior to recruitment, at the first 3 echocardiography timepoints and prior to outcome assessment. A schematic of the timeline is presented in Figure 2.

Echocardiography scans are performed at five time periods: between 36 – 48 hours (for enrolment), following the first course of medication; following the second course of medication if indicated, at 36 weeks corrected age, and at 3 months corrected age. The first 4 scans are performed at the bedside and the final scan is performed in an outpatient setting. Evaluations will be performed using the Vivid S6 (GE Medical, Milwaukee) echocardiography system in accordance with recent published guidelines ^40^. The first echocardiogram is a detailed anatomic assessment to out rule congenital heart disease other than a patent foramen ovale (PFO) or PDA. All echocardiography studies are safely stored in an offline archive. Offline measurements are performed away from the bedside at a later stage.

 “A comprehensive echocardiography assessment of PDA characteristics, markers of pulmonary overcirculation and systemic hypoperfusion, and LV function will be performed. The following echocardiography measurements will be obtained during each assessment [description of the methodology used to obtain those measurements are detailed elsewhere] ^40– 42^: narrowest PDA diameter (mm) measured using 2D methods at the pulmonary end (colour Doppler will not be used to assess PDA diameter); maximum shunt velocity across the PDA (Vmax in m/s); left ventricular output (LVO in ml/kg/min); mitral valve inflow E wave, A wave, and E:A ratio; pulmonary vein diastolic velocity (PVd in m/s); left atrial to aortic root ratio (LA:Ao); descending aortic, celiac artery and middle cerebral artery (MCA) end diastolic flow (EDF in m/s). Tissue Doppler imaging (TDI) of the apical 4-chamber view was used to obtain LV systolic (s`) early diastolic (e`) and late diastolic (a`) velocities using a pulsed wave Doppler sample gate of 2 mm at the level of the lateral mitral valve annulus”. This echocardiography assessment is cited from the methodology section of a previously published article by our research group ^29^ Speckle tracking echocardiography to obtain strain and rotation parameter of the LV. A complete set of echo measurements is summarised in Appendix I which is available to review as extended data ^43^.

### Sample size

There are approximately 90 infants, ≤ 29 weeks gestational age at birth, admitted to the Rotunda NICU annually. We anticipate a 50 – 70% enrolment rate per year to account for consent refusal, competition with other studies, exclusions and unavailability of staff to recruit patients. Therefore, the original anticipated recruitment period was 12 – 18 months. The anticipated end date for the study has been amended to 31-Dec-2019 due to delays in recruiting. This study is conducted to determine the sample size necessary for a definitive multicentre trial. A sample of 30 infants per arm (a total of 60 infants) will be recruited over the recruitment period.

This is a feasibility study and the sample size was selected based on the above reasoning. However, the sample will be sufficient to demonstrate a significant difference in the primary outcome between the groups if the event rate is reduced from 90% in the control arm to 55% in the intervention arm. Prior data indicate that the CLD/Death amongst controls is 90%. If the CLD/Death rate for experimental subjects (treated infants) is 55% we will be able to reject the null hypothesis with a probability (power) of 80% based on a sample size of 24 per arm. The Type I error probability associated with this test of this null hypothesis is 0.05. We will use an uncorrected chi-squared statistic to evaluate this null hypothesis. We will use an uncorrected two tailed chi-squared statistic to evaluate this null hypothesis.

### Recruitment

All babies admitted to the Rotunda NICU with a gestation at birth <29 weeks will be considered for recruitment.

## Methods: Assignment of interventions (for controlled trials)

### Allocation

A computer-generated central randomisation scheme will be used to assign the infants to the two arms in a 1:1 ratio. Infants will be stratified into two gestational age brackets 23 – 26 weeks and 27 – 28 weeks. The study pharmacist will receive a binder containing the sequence of treatment group assignments for the cohort from a statistician who will not otherwise be involved in the study. Access to the binder will be restricted to selected pharmacy personnel, and will be kept securely in the pharmacy department.

### Blinding (masking)

Study treatment assignment will be blinded for both the investigators and the subject. Once a patient is recruited and randomised to either Ibuprofen or Placebo, then a designated unblinded trial pharmacist who is not involved in recruitment, allocation, and data collection will prepare the trial drug or placebo and issue the syringe for infusion to the trial investigator team for administration. The Ibuprofen preparation is colourless and odorless and will be indistinguishable from the saline preparation used for the placebo arm. The trial participants and their families, the care providers, the data collectors, the echocardiographers, the primary outcome assessors, and the data analysts will all be blinded to the allocation. Blinding will be unmasked if serious adverse events arise.

## Methods: Data collection, management, and analysis

### Data collection methods

Source documents for this study will include hospital records, nursing vital monitoring chart, and the echocardiography archiving system. These documents will be used to enter data on the CRFs. All data entered on CRFs must be entered legibly. If an error is made, the error will be crossed through with a single line in such a way that the original entry can still be read. The correct entry will then be clearly inserted, and the alterations will be initialled and dated by the investigator.

Data reported on the CRF that are derived from source documents must be consistent with the source documents, or the discrepancies must be explained. All documents will be stored safely in confidential conditions. On all study-specific documents other than the signed consent, the subject will be referred to by their unique study participant identification number/code.

### Data management

Data will be entered numerically or as a dichotomous variable. See Appendices II-IV for data entry form samples which is available to view as extended data ^43^. The subjects will be identified by a study specific subject number in the database. The name and any other identifying detail will not be included in any study data electronic file. No individual patient data will be presented. All data will be presented summarized as means, medians, or proportions as appropriate

Direct access to the data will be granted to authorised representatives from the Sponsor, host institution and the regulatory authorities to permit trial-related monitoring, audits and inspections. Clinical and demographic data, as well as outcome data will be collected by the research fellow who will be blinded to treatment allocation. Pilot testing of the data collection sheet will be performed prior to study commencement. No patient identifiers will be used and each patient will be assigned a unique ID number based on the sequence in recruitment.

The Investigator will retain the essential documents relating to the trial for a minimum period of 5 years after its completion as per local regulatory requirements, or for a longer period where so required by an agreement with the Sponsor. The Sponsor will inform the Investigator when these documents are no longer necessary and direct disposition

### Statistical methods

The primary outcome, and most of the secondary outcomes, are dichotomous variables. Continuous variables will be tested for normality by comparing the mean and median, a histogram representation of data, and the Shapiro-Wilk test for normality and will be presented as means (standard deviation: SD) or median [inter-quartile range] as appropriate. Dichotomous variables will be presented as proportions and summarised in contingency tables. A chi squared test will be used for the primary analysis of the dichotomous primary and secondary outcomes. Fisher’s exact test (in place of the chi-squared test) will be used when the counts in one or more cells have an expected frequency of five or less (for 2 by 2 table). For the continuous secondary outcomes, an independent samples t-test will be used to compare normally distributed data, and Wilcoxon Rank Sum test will be used for skewed data. All tests will be two sided and we will accept a p value of < 0.05 as statistically significant. We will use IBM SPSS ^®^ (Version 22) to perform the statistical analysis. All enrolled infants will be analysed on an intention-to-treat basis. Analysis for the feasibility study will only be conducted once the recruitment of all patients is completed. No interim analysis of treatment effect will be conducted.

## Methods: Monitoring and regulatory issues

The study will be conducted in accordance with the approved protocol, the ICH Harmonised Tripartite Guideline for Good Clinical Practice, relevant regulations and standard operating procedures (SOPs) ^44^. Trial sponsorship and monitoring will be carried out by the Royal College of Surgeons in Ireland (RCSI) whose responsibility will be to conduct internal audits, ensure correct implementation of SOPs, record protocol deviations, and initiate procedures to correct any shortcomings and prevent their recurrence. The safety of the investigational medicinal product (Ibuprofen) will be assessed through the recording, reporting and analysing of baseline medical conditions, adverse events, vital signs, physical exam findings, and laboratory tests in line with good clinical practice (GCP). A Trial Management Group (TMG) will be set up to be responsible for integration of the study protocol within the clinical setting, training of study personnel, adherence to study protocol and maintenance of concealment and adequate randomisation. An independent data safety and monitoring board (DSMB) will assess the quality and timeliness of data collection and review the safety data at regular intervals. Unblinding of allocation will be conducted after the enrolment and blinded data collection at 36 weeks corrected age of the final recruited infant. The trial was registered with ISRCTN ( ISRCTN13281214) on the 26 ^th^ July 2016 and the European Union Drug Regulating Authorities Clinical Trials Database ( 2015-004526-33) on the 3 ^rd^ March 2015.

### Harms

The safety of the investigational medicinal product (Ibuprofen) will be assessed through the recording, reporting and analysing of baseline medical conditions, adverse events, vital signs, physical exam findings, and laboratory tests in line with good clinical practice (GCP). The study investigators will adhere to all protocols, safety procedures and standard operating procedures (SOPs) as set out by the Sponsor (RCSI). All adverse events occurring from consent until the 120—168 hour visit will be collected and recorded in the CRF.

## Ethics and dissemination

### Research ethics approval

Ethical approval was received from the research ethics committee for RCTs at the National Maternity Hospital on the 05 ^th^ of September 2016 (EC09.2016). HPRA approval of the trial was sought prior to commencement of the study and was received on January the 29 ^th^ 2016 (CT 900/577/1). Any amendments to the protocol will be submitted to both the HPRA and the research ethics committee. The Sponsor will ensure that this study is conducted in accordance with the ethical principles that have their origins in the Declaration of Helsinki. This study will be conducted in accordance with Good Clinical Practice (GCP), as defined by the International Conference on Harmonisation (ICH) and in accordance with the ethical principles underlying European Union Directive 2001/20/EC and 2005/28/EC.

The local PI will initially approach the physician responsible for the care of the infant for permission to approach the parents with a view to informing them of the pilot study. Parents of eligible infants will be approached over the first 36 hours of age to obtain written informed consent before carrying out the echocardiogram (ECHO). All parents will be provided with a clear explanation of the objectives, procedures, risks and benefits of the study in the Patient Information Leaflet (PIL). The investigator must provide time and opportunity for them to ask questions and ascertain details of the study. Further information will be given verbally and all questions will be answered. Parents will be given 12 hours to consider enrolment into the study. Once all discussions are completed, they will be invited to sign the consent form.

Informed consent will be obtained before any study assessments/ procedures are performed and before any data collection occurs. All personal study participant data collected and processed for the purposes of this study will be managed by the investigators and their staff with adequate precautions to ensure the confidentiality of those data and in accordance with applicable national and local laws and regulations on personal data protection. The ethics committees approving this research will be granted direct access to the study participants’ original medical records for verification of clinical trial procedures and/or data, without violating the confidentiality of the participants, to the extent permitted by the law and regulations.

## Dissemination of information

The findings and data of our study will be disseminated through presentation at national and international paediatric and neonatal conferences. We will also endeavour to publish our findings and study data in peer reviewed research journals.

## Study status

Recruitment for the trial finished in January 2017 when the 60 ^th^ baby was randomised. Unblinding of allocation was conducted after the enrolment and blinded data collection at 36 weeks corrected age of the final recruited infant. Data has been prepared for publication in a peer reviewed research journal and for presentation at an international paediatric and neonatal conference.

## Conclusion

### Project long-term sustainability

This project potentially fulfils the funding strategies of the national funding agencies with increased collaboration between academic and research funding institutions. It fosters international collaboration and facilitates knowledge translation. This study also expands the research into diseases of vulnerable population.

### Relevance of primary outcome to patients and parents

Chronic lung disease (CLD) is an extremely serious complication of premature delivery associated with high mortality rates both before and after discharge. In addition, CLD is associated with adverse neurodevelopmental outcome, poor growth and significant respiratory morbidity, with reduced lung function demonstrated into adulthood ^30^. Necrotizing enterocolitis affects long term bowel function (including the presence of a stoma, admissions for bowel related problem and continued medical and surgical care) as well as impacting on motor, sensory and cognitive outcomes ^31^.

### Significant savings for the health service

A positive outcome in this pilot study will pave the way for a definitive trial of targeted PDA treatment. Reducing PDA-associated complications such as necrotizing enterocolitis and chronic lung disease will have substantial short and long term benefits for the healthcare service. Reduced length of stay will significantly reduce hospital costs in the neonatal intensive care unit. Ongoing management of NEC and CLD in the short and longer term poses a huge financial burden on the health service. In addition, NEC and CLD increase the risk of adverse neurodevelopmental outcome with all its associated ongoing financial and quality of life costs.

### Reducing Burdens on Families

Reducing the rate of CLD and NEC will have substantial short and long term benefits for the infants and their families. Reducing hospital stay, improving the response rate and reducing the chance of undergoing surgery will improve the quality of life of the infant, facilitate earlier nutrition and discharge, and potentially improve neurodevelopmental outcome.

### Establishing the Irish Neonatal Hemodynamic Program as a global leader in cardiovascular health research and newborn care delivery

An international network of Irish neonatologists in Ireland and Canada have garnered a global reputation in leading innovative neonatal hemodynamic research based on defining actual physiology, understanding mechanism of disease and defining populations of interest prior to embarking on treatment trials. This philosophical approach formed the basis for studies designed to characterize post-PDA ligation physiology, early hemodynamic significance of the PDA and the physiology of complex hemodynamic situations such as Persistent Pulmonary Hypertension of the Newborn (PPHN). This pilot study will be the first randomised controlled trial to evaluate the use of targeted PDA treatment based on a risk score. This study, along with several other HRB IP CTN trials led by this group (the HIP Trial which evaluates blood pressure support in preterm infants, again the first of its kind; and the MINT trial- examining the role of Milrinone in the treatment of persistent pulmonary hypertension of the newborn) will consolidate this Irish collaborative research network as world leaders in the area of newborn cardiovascular support. 

### Enhanced collaboration and consortium building for H2020

This pilot study will build on the already established HRB IP CTN links across many European centres and will form the basis of a Horizon 2020 bid in 2016.

## Data availability

### Underlying data

No data are associated with this article

### Extended data

Figshare: Early Targeted Patent Ductus Arteriosus Treatment in Premature Neonates Using a Risk Based Severity Score: Appendices.docx. https://doi.org/10.6084/m9.figshare.13058228.v1 ^43^

This project contains the following extended data:

- PDA RCT Appendices.docx (The appendices include the following: appendix I: data echocardiography measures; appendix II: eligibility checklist and parameters during echocardiography; appendix III: approach to confirming normal structural anatomy; appendix IV: data collection sheet)

### Reporting guidelines

Figshare: SPIRIT checklist for ‘Early targeted patent ductus arteriosus treatment in premature neonates using a risk based severity score: study protocol for a randomised controlled trial (PDA RCT)’ https://doi.org/10.6084/m9.figshare.13058228.v2 ^43^
