# Supplementary material for: Early targeted patent ductus arteriosus treatment in premature neonates using a risk based severity score: study protocol for a randomised controlled trial (PDA RCT)
Source: HRB Open Res. 2020 Nov 25;3:87. [Version 1] doi: 10.12688/hrbopenres.13140.1 (PMC8422343; doi:10.12688/hrbopenres.13140.1)
Supplement: Peer review history

# Peer review: Reviewer response for version 1

**DOI:** 10.21956/hrbopenres.14253.r30183

**Published:** 2021-09-06

**Authors:** Yung-Chieh Lin, Jieh-Neng Wang

**Affiliations:** 1 Department of Pediatrics, National Cheng Kung University, Tainan, 70457, Taiwan; 2 Department of Pediatrics, National Cheng Kung University, Tainan, Taiwan

In this reported protocol for a randomized trial for PDA medical treatment, the authors conducted the trial based on a PDAsc developed from their publication (ref. 29; PubMed ID 24674706). The score was calculated by 5 variables independently associated with CLD/death (gestation at birth, PDA diameter, maximum flow velocity, LV output, and LV a' wave). The authors will enroll infants with the score ≥ 5.0 and started the randomized trial if no exclusion criteria matched. The protocol is well designed and completely considered many clinical scenarios. Backup therapy was provided. The protocol is feasible could be conducted in other NICU.

 Here are my comments and suggestions:

**Introduction:**

- The background rationale was clearly described.  To date, PDA closure may be also related to the improvement of pulmonary score not only in baboon but also in human (PMID 34063345; PMID: 30269408).

**Methods:**

- One point of exclusion criteria should be confirmed again. The definition of thrombocytopenia was platelet count < 100/mm3. Is it not too low?
- Do the author consider to follow-up infants with PDA and the score < 5.0 (and without major exclusion criteria) as the same protocol? This population will be interesting to many readers.

The appendices document was also clearly presented.

Is the study design appropriate for the research question?

Yes

Is the rationale for, and objectives of, the study clearly described?

Yes

Are sufficient details of the methods provided to allow replication by others?

Yes

Are the datasets clearly presented in a useable and accessible format?

Yes

Reviewer Expertise:

Neonatology. Critical pediatric care.

We confirm that we have read this submission and believe that we have an appropriate level of expertise to confirm that it is of an acceptable scientific standard.

# Peer review: Reviewer response for version 1

**DOI:** 10.21956/hrbopenres.14253.r29120

**Published:** 2021-05-04

**Authors:** Sanket Shah

**Affiliations:** 1 Department of Pediatrics, Division of Neonatology, University of Florida College of Medicine, Jacksonville, FL, USA

The author has explored an important and highly debated question in extremely premature infants. As authors have mentioned the importance of PDA in preterm infants. PDA severity score was an innovative idea and authors have shown data in their previous study. This study is to aim to identify infants at high risk of developing CLD/Death and treatment for PDA / placebo. I congratulate authors for conducting a study and using PDA severity score. The study is designed very well and narrated in great details.

 Here are my comments and suggestions.  

1. **Introduction: **
  - Please provide a reference to "At this stage closure of the DA will usually occur as it is no longer required. However, if the DA remains open following birth, especially in the premature population, it is associated with important pathological effects".
  - Provide a reference to “The ductus arteriosus plays an important role during the transition from intra-uterine life in the premature babies’ early hours and as such it should be viewed as a beneficial physiological component in aiding the reduction of pulmonary hypertension and supporting the right ventricle, as pulmonary vascular resistance drops.”
2. **Methods: **

- **Study Design**
  - Please define hypotension on line 6.
- **Overdose of study treatment: **
  - Please describe normal range of blood pressure, heart rate, and urine output for preterm infants.

Is the study design appropriate for the research question?

Yes

Is the rationale for, and objectives of, the study clearly described?

Yes

Are sufficient details of the methods provided to allow replication by others?

Yes

Are the datasets clearly presented in a useable and accessible format?

Yes

Reviewer Expertise:

Neonatology

I confirm that I have read this submission and believe that I have an appropriate level of expertise to confirm that it is of an acceptable scientific standard.
